# Factors influencing Generation Z’s intention to purchase sustainable clothing products in Vietnam

**DOI:** 10.1371/journal.pone.0315502

**Published:** 2024-12-11

**Authors:** Thi Thuy An Ngo, Chi Hai Vo, Ngoc Lien Tran, Khanh Vy Nguyen, Thanh Dat Tran, Yen Nhi Trinh

**Affiliations:** 1 Department of Soft Skills, FPT University, Can Tho City, Vietnam; 2 Department of Business, FPT University, Can Tho City, Vietnam; Krirk University, THAILAND

## Abstract

The increasing awareness of environmental challenges has significantly influenced consumer behavior, with sustainable products, particularly sustainable clothing, becoming a top priority for Generation Z consumers. This study aims to investigate the factors influencing Generation Z’s purchase intentions toward sustainable clothing in Vietnam, guided by the Stimulus-Organism-Response (SOR) model and Theory of Planned Behavior (TPB) frameworks. Specifically, it examines the effects of green perceived value, green perceived quality, perceived price, social influence, product design, environmental concern, and environmental knowledge on purchase intentions. The study also explores the mediating roles of environmental attitude and product attitude in these relationships. The research employed a quantitative approach, using a Likert scale questionnaire to gather data from 641 Vietnamese Generation Z consumers. The study utilized exploratory factor analysis (EFA), confirmatory factor analysis (CFA), and structural equation modeling (SEM) to analyze the data. The results revealed that all proposed hypotheses were supported, indicating that all factors significantly impact environmental attitude and product attitude, which, in turn, influence purchase intention. The results emphasize the strong mediating role of environmental attitude and product attitude, suggesting that consumers with positive attitudes toward the environment and products are more likely to intend to purchase sustainable clothing. This research provides valuable insights into the psychological and contextual factors that influence Generation Z’s sustainable consumption behavior. For marketers, these findings underscore the need to promote transparency in sustainable practices, emphasize high product quality and appealing designs, and engage this demographic through community involvement and authentic sustainability efforts.

## 1. Introduction

The Earth is currently facing the most severe environmental challenges ever, with climate change emerging as a pressing issue according to recent reports from the Intergovernmental Panel on Climate Change [1]. Economic activities, particularly in the fashion industry, have been identified as significant contributors to these crises. Despite its glamorous facade, the fashion industry stands as one of the largest polluters globally, responsible for substantial carbon emissions and environmental degradation. It contributes 10% of total carbon emissions worldwide, second only to the oil industry in terms of pollution [2]. This industry’s manufacturing processes are notorious for their intensive water use, chemical emissions, and high energy consumption, which exacerbate environmental concerns [3, 4]. Hazardous waste generated during production poses additional threats to the environment, including soil contamination and water pollution [5]. A major threat is imposed on the environment due to the discharge of untreated toxic wastewater into water bodies that can create severe damage to the aquatic ecosystems [6]. In many developing nations, including Vietnam, the textile industry ranks as the second most polluting sector after the oil industry [7]. However, despite its critical environmental impact, sustainability issues within the fashion industry are often neglected in efforts toward more sustainable development [8]. Whitmarsh et al. [9] have emphasized that targeting large-scale emitters and addressing high-impact consumer behaviors is critical for tackling climate change. Excessive clothing consumption, driven by fast fashion, contributes heavily to waste and resource depletion, hindering the industry’s transition to a sustainable bioeconomy [10]. This aligns with the theory of sufficiency, which argues that simply offering greener products is not enough to drive systemic change. Instead, a fundamental shift away from overconsumption is needed to promote long-term, sustainable solutions for environmental issues [11, 12].

The rise of fast fashion, driven by a ‘throwaway society’ has accelerated the environmental toll of the fashion industry. The exponential increase in fashion production since the 2000s has led to extreme resource wastage and a culture of impulse buying [13, 14]. This situation becomes even more severe in developing economies like Vietnam, where rapid industrial growth is accompanied by significant environmental degradation, including air and water pollution [15]. Vietnam’s textile and garment industry, a cornerstone of the national economy, plays a key role in this dynamic. With annual exports valued in the billions [16, 17], the industry has brought substantial economic benefits but also contributed to Vietnam’s escalating environmental challenges. Vietnam ranks among the world’s largest textile exporters, and this position comes with high production demands that frequently exacerbate resource overconsumption, chemical waste, and carbon emissions. These factors, combined with the scarcity of sustainable practices in some areas, have intensified concerns over the industry’s environmental footprint.

In response to these environmental concerns, the fashion industry has witnessed a growing movement toward sustainable practices, with Generation Z emerging as a key demographic advocating for eco-friendly consumption. This cohort differs from previous generations due to their heightened awareness and inclination to adopt sustainable products [18, 19]. Research has investigated various factors influencing eco-friendly purchase behaviors, focusing on values such as environmental attitudes, knowledge, pricing, and social influence [20, 21]. Camilleri et al. [21] highlighted that consumers evaluate sustainable products not only based on price and quality but also on social influence and emotional value. Additionally, environmental concerns, attitudes, and social norms influence consumers to adopt sustainable practices, including actions such as donating old clothes to charities or giving them to family members and friends [22]. However, there remains a critical gap in the literature regarding Generation Z’s sustainable consumption behaviors in developing countries like Vietnam. With its predominantly young population and an emerging, environmentally conscious Generation Z, Vietnam offers a unique context to investigate sustainable clothing purchase intentions. Understanding the extent to which Vietnamese Generation Z consumers consider environmental issues in their purchasing decisions is essential for gauging the potential for sustainable consumption in one of the fastest-growing fashion markets globally. While studies have explored this cohort’s sustainable clothing consumption in other regions like Peru, Australia, UK [23–25], the specific cultural and economic context of Vietnam has not been thoroughly examined. This gap is particularly relevant given that Vietnam represents a distinctive market where rapid economic development intersects with increasing environmental awareness.

This study is primarily grounded in the Stimulus-Organism-Response (S-O-R) model, which explains how external factors like green perceived value, social influence, and environmental concern (stimuli) affect consumers’ internal states, represented by environmental and product attitudes (organism), and how these attitudes influence purchase intention (response). Additionally, it draws on the attitude component of the Theory of Planned Behavior (TPB), which posits that attitudes toward a behavior are key drivers of intentions. By integrating these frameworks, the study provides a comprehensive understanding of the factors shaping Generation Z’s sustainable clothing purchase intentions in Vietnam. The inclusion of these variables is theoretically supported by prior research. For instance, Yadav and Pathak [26] highlight the importance of environmental concern and knowledge in shaping consumer behavior. Studies by Chen et al. [27] have identified the roles of product attitude and environmental attitude as mediators between independent variables and purchase intention, while social factors, such as social influence, have been explored by Purboyo [28]. Moreover, van der Merwe [29] research on product design has shown its crucial role in influencing consumer preferences. Recent research by Liao et al. [30] and Jin et al. [31] has also examined how perceptions and attitudes affect purchasing intentions. Yet, no study has comprehensively examined these factors within a single framework, particularly in Vietnam, a fast-growing economy with rising sustainability concerns.

This study addresses the theoretical and practical gap in understanding Generation Z’s sustainable clothing consumption in Vietnam, combining variables that have been previously studied in isolation. The novelty of this research lies in its holistic approach, integrating green perceived value, green perceived quality, perceived price, social influence, product design, environmental concern, and environmental knowledge to examine how these factors shape environmental and product attitudes and ultimately influence purchase intention. Moreover, the study leverages the Vietnamese cultural context, where rapid industrial growth coexists with emerging environmental consciousness, to offer new insights into sustainable consumption patterns. The findings offer significant practical implications for marketers, policymakers, and fashion industry stakeholders. By understanding the distinct motivations and attitudes of Generation Z in Vietnam, stakeholders can develop targeted strategies to promote sustainable fashion, foster eco-friendly consumption behaviors, and address pressing environmental issues. Furthermore, this study contributes valuable insights into the complex interplay between economic growth, consumer behavior, and environmental sustainability, guiding sustainable practices within Vietnam’s fashion industry. These insights also offer broader implications for other developing economies facing the dual challenges of industrial expansion and environmental sustainability, supporting efforts to balance these goals effectively.

## 2. Literature review

### 2.1. Theoretical background

#### 2.1.1. The Stimulus-Organism-Response (S-O-R) model

The Stimulus-Organism-Response (S-O-R) model, originally developed by Mehrabian and Russell [32], proposes that environmental stimuli influence an individual’s emotional (affective) and cognitive (perceptual) reactions, which subsequently shape their behavior. The model suggests that stimuli serve as external triggers that arouse consumers, leading to internal emotional and cognitive responses that ultimately determine behavioral outcomes [32, 33]. This theoretical framework has become widely used in consumer behavior research to explain how various external factors influence consumer decision-making.

The first element of the model, stimuli, refers to external factors that trigger consumer responses. Stimuli can encompass marketing efforts, such as product features, advertising, or situational factors, including social or environmental conditions that provoke consumer interest [34, 35]. In the context of this study, the stimulus includes factors like green perceived value, green perceived quality, perceived price, social influence, product design, environmental concern, and environmental knowledge. These stimuli are external motivators that encourage consumers to consider purchasing sustainable products. As noted by Kotler and Keller [36], these stimuli help raise consumers’ awareness of eco-friendly products, shaping their perception of value and quality, thereby influencing their purchase intentions.

The second component, the organism, represents the internal cognitive and affective processes that mediate between the external stimuli and the eventual behavioral response. These internal states can include emotional reactions or cognitive evaluations, which influence how consumers process the stimuli they encounter [37]. In this study, the organism is represented by environmental attitude and product attitude. Environmental attitude reflects consumers’ concerns and overall awareness of environmental issues. Consumers with positive environmental attitudes are more inclined to purchase green products, as their values align with eco-friendly practices [38]. Product attitude refers to consumers’ evaluation of the product’s quality and perceived value. When consumers hold positive attitudes toward green products, they are more likely to engage in sustainable purchasing behavior [39]. These internal attitudes are crucial in shaping how individuals respond to the stimuli they encounter.

Finally, the response refers to the consumer’s behavioral outcome following their internal processing of stimuli. In this model, the response often manifests as a behavioral tendency toward either approach (engagement) or avoidance (rejection) of the product or behavior [35]. For this study, the response is represented by purchase intention, which indicates the likelihood of a consumer purchasing a sustainable product. According to Ajzen [40], purchase intention is one of the most reliable predictors of actual consumer behavior. It is the outcome of both external stimuli and the consumer’s internal evaluation process. In the context of this research, purchase intention reflects a consumer’s willingness to buy eco-friendly clothing after being influenced by external environmental factors and their internal attitudes toward both the environment and the product.

The S-O-R model has been applied extensively to studies on consumer behavior, particularly within the realm of sustainability. For instance, Guo et al. [41] highlight that the unique features of online shopping, as external stimuli, evoke emotional and cognitive responses in consumers, leading to either an intention to purchase or an avoidance of specific products. In this study, the model is used to explore how external stimuli, such as product quality, price, social influence, and environmental concern, interact with internal factors like attitudes to drive the intention to purchase sustainable clothing products. This theoretical approach provides a comprehensive framework for understanding how consumers navigate their decisions in the context of sustainable consumption, emphasizing the interaction between external market stimuli and internal psychological factors.

#### 2.1.2. Theory of Planned Behavior (TPB)

The Theory of Planned Behavior (TPB) builds on the Theory of Reasoned Action (TRA) by adding perceived behavioral control, addressing TRA’s limitation of focusing only on attitude and subjective norms. This addition makes TPB a more comprehensive psychological theory, capable of explaining not just behaviors within an individual’s full control, but also those constrained by external factors [40].

The attitude toward behavior refers to an individual’s positive or negative evaluation of performing a specific action. This attitude significantly impacts the likelihood of forming an intention to carry out the behavior, with more favorable attitudes increasing the probability of intention formation [42, 43]. Subjective norms, on the other hand, involve societal pressures and the expectations of others, which can either encourage or discourage an individual from intending to engage in a behavior [40]. The final component, perceived behavioral control, reflects the degree to which individuals believe they can successfully perform a given behavior. This belief is shaped by perceived opportunities, resources, and potential barriers, all of which influence their decision-making process [40].

TPB is particularly valuable in research related to behaviors that require careful consideration of external factors, such as energy-saving practices and the adoption of environmentally friendly products. This theory provides a framework for understanding the underlying psychological processes that drive consumer decision-making, especially in contexts like sustainable product consumption [44]. In the context of this study, the focus is on the attitude component of TPB to build a research model that examines the purchase intentions for sustainable clothing among Generation Z in Vietnam.

The central role of attitude in shaping behavior has been widely acknowledged in previous studies. For instance, Ahmed et al. [45] applied TPB to explore how attitudes toward green products influence green purchase intentions. Similarly, this study uses attitude as a key variable to provide a deeper understanding of how it mediates the relationship between external factors and the intention to purchase sustainable clothing. By focusing on attitudes, the study aims to capture the internal dispositions that influence Generation Z’s inclination to engage in environmentally conscious purchasing decisions in Vietnam. Through this approach, the research contributes to a broader comprehension of the factors that shape consumer behavior in the context of sustainability.

#### 2.1.3. Sustainable clothing

Sustainable clothing has been defined as apparel that integrates elements of social and environmental sustainability, such as Fair-Trade practices and the use of organic materials [46]. While sustainable clothing offers a promising solution to the environmental harm caused by traditional garment manufacturing processes [47], its adoption is not without challenges. One major issue is the attitude-behavior gap, where consumers express a favorable attitude toward sustainable products but do not consistently translate these attitudes into actual purchase behavior [48]. This discrepancy often arises from factors like higher pricing, limited availability, or skepticism about the environmental claims made by brands [49]. Studies suggest that while many consumers, particularly in regions like Germany and the U.S., consider sustainability an important purchase criterion [50, 51], there is still a large portion of the market resistant to changing consumption patterns, especially in countries where fast fashion dominates consumer preferences.

Moreover, despite the increased awareness around sustainability, the fashion industry continues to contribute significantly to environmental degradation, accounting for approximately 10% of global carbon emissions [2]. This suggests a need for stronger consumer education and advocacy, as well as industry-wide shifts towards sustainable production practices [49, 52]. Scholars have suggested that collaborative consumption models—such as clothing exchanges and second-hand markets—can help reduce waste and extend the life cycle of clothing products, mitigating the impact of overconsumption [53]. However, a critical challenge lies in balancing consumer demand for affordability and style with sustainability goals. Although sustainable product attributes (i.e., recycled materials) have been shown to positively influence consumers’ purchase intentions even for luxury and fast fashion products [54]; there is skepticism toward corporate brands, with some consumers doubting the authenticity of their sustainable claims, a phenomenon sometimes referred to as “greenwashing” [55–57]. This highlights the need for transparent communication from brands and third-party certifications to build trust among consumers.

#### 2.1.4. Generation Z

Generation Z, defined as those born between 1997 and 2012 [58], has been extensively studied for their unique purchasing habits and their significant role in driving sustainability within the fashion industry [16, 59, 60]. This demographic shows a strong sense of social responsibility, with approximately 59% expressing genuine concern for environmental issues and supporting sustainable consumption [61].

Despite their strong understanding of the importance of ecological friendliness, Generation Z often encounters challenges in translating this awareness into actionable involvement [60]. However, their distinct inclination towards adopting eco-friendly products sets them apart from previous generations [19]. Notably, Generation Z integrates sustainability into their broader lifestyle choices and actively adopts eco-friendly products, making them a highly influential market segment [62, 63]. Referred to as “guardians of sustainability” by PricewaterhouseCoopers (PwC), their commitment to sustainability positions them as key drivers in promoting sustainable practices [64].

In conclusion, Generation Z’s significant influence on the fashion industry, combined with their commitment to sustainable practices, underscores their unique position in driving the shift towards sustainability. Their purchasing habits, informed by a strong sense of social responsibility and a willingness to adopt eco-friendly practices, make them a critical demographic for marketers and businesses aiming to promote sustainable consumption.

#### 2.1.5. Purchase Intention (PI)

Purchase intention (PI) refers to the cognitive process through which consumers evaluate and decide whether to buy a specific product or service. This evaluation is influenced by various factors, including individual preferences, tastes, past experiences, and the perceived attributes of the product, such as quality, price, and brand reputation [65]. Due to its significant role in the decision-making process, purchase intention has been widely recognized as a key factor for understanding, predicting, and influencing consumer behavior [66]. It serves as an indicator of the likelihood that a consumer will engage in a purchase, thus acting as a crucial bridge between the awareness of a product and the actual act of buying. Research by Nabilla [67] also emphasizes that a sustained purchase intention can lead to consistent consumer behavior over time, indicating that when consumers are committed to the idea of purchasing a product, they are more likely to follow through with their intentions. This commitment can manifest in various ways, such as actively seeking information about the product, comparing alternatives, and ultimately making a purchase. However, in the context of ethical consumerism, the extent to which purchase intention translates into actual purchase behavior is not well understood [48]. Despite consumers often expressing a desire to purchase eco-friendly clothing, this intention frequently does not translate into corresponding buying behaviors [68, 69]. Several factors contribute to this disconnect, including income constraints, convenience issues, and perceptions of product quality. Understanding these barriers is vital for manufacturers and retailers aiming to develop effective strategies to encourage the purchase of sustainable products [49]. Moreover, studies have shown that Generation Z increasingly embraces sustainable lifestyles, favoring durable, high-quality products [70]. This demographic demonstrates a willingness to pay more for sustainable options and actively supports companies that prioritize sustainability, while also being prepared to boycott those that are perceived as unsustainable [71]. Therefore, understanding Generation Z’s sustainable clothing purchasing behavior is crucial for developing effective, targeted marketing strategies for this influential consumer group.

### 2.2. Research framework and hypotheses development

#### 2.2.1. Green Perceived Value (GPV) and consumer attitude

Green perceived value (GPV) is a consumer’s perception of the value of a good or service based on its environmental benefits. This concept includes consumers’ environmental consciousness and their preference for products with sustainable attributes [72]. In the context of sustainable clothing, green perceived value refers to the perceived value derived from purchasing and utilizing products that contribute to environmental well-being [73]. GPV represents a balance between the value consumers receive from sustainable clothing, such as environmental conservation, health benefits, and the cultivation of sustainable lifestyle habits, and the associated costs [74]. It broadens the traditional understanding of perceived value by integrating environmental considerations into consumer evaluations. As a result, consumers do not merely assess the conventional “profit and loss” of a product; they also take its environmental impact into account [75]. This enhanced perception of value significantly influences consumer behavior by altering how eco-friendly products or services are viewed, thereby increasing their overall attractiveness in the consumer’s mind. Similar to general perceived value, green perceived value affects brand perception and attitude, as well as purchasing decisions [76]. Consumers with a strong commitment to environmental issues are more inclined to select brands that align with their sustainability values, thereby reinforcing brand loyalty and influencing their long-term purchasing behavior.

Research by Yadav and Pathak [26] explored the relationship between green perceived value and the intention to purchase sustainable clothing, with findings suggesting that a green consumption attitude serves as a mediating factor. Their findings suggest that consumers’ attitudes towards green products significantly influence their purchasing intentions. This aligns with insights from Liao et al. [30], who found that green perceived value significantly shapes consumer behavior by influencing attitudes towards green products. Moreover, the perceived value of green products enhances consumers’ sense of responsibility and their focus on higher levels of green product branding [30]. This implies that when green brand positioning is emphasized, customers are more likely to purchase green products. Therefore, a well-established green brand positioning can lead to higher consumer intentions to buy green products. These studies collectively underscore the pivotal role of GPV in shaping consumer behavior toward sustainable products. It influences not only attitudes and purchasing intentions but also enhances consumers’ sense of responsibility for environmental conservation. Based on these literature, the following hypotheses are proposed:

**H1:** Green perceived value significantly affects environmental attitude.
**H2:** Green perceived value significantly affects product attitude.

#### 2.2.2. Green Perceived Quality (GPQ) and consumer attitude

Green perceived quality (GPQ) represents customers’ judgment of a product’s overall quality or excellence concerning its intended purpose, relative to alternative options [77]. It is defined as the overall assessment of a product’s environmental superiority or excellence by customers [78]. Green perceived quality serves as a distinct attribute that significantly influences evaluations among green consumers Alamsyah et al. [79] and is a critical determinant for producers and marketers as it creates an opportunity for product differentiation [80, 81]. This encompasses aspects such as product performance, usability, reliability, and durability.

Empirical evidence underscores the effectiveness of green perceived quality in eliciting positive responses from customers and fostering purchase intentions [78]. Research results have shown that the higher consumers’ perception of green quality, the higher their attitude towards the environment, thereby leading to increased purchase intention. Additionally, research by Chen et al. [82] highlights the statistically significant and positive relationship between environmental attitude, product attitude, and perceptions of green product quality. Studies indicate that improving green perceived quality will lead to increased customer trust in green products, thereby fostering better consumer attitudes towards these products. This relationship underscores the importance of green perceived quality in influencing consumer behavior and highlights its role in promoting sustainable consumption. Building upon these insights, the following hypotheses are proposed:

**H3:** Green perceived quality significantly affects environmental attitude.
**H4:** Green perceived quality significantly affects product attitude.

#### 2.2.3. Perceived Price (PP) and consumer attitude

Perceived price (PP), as defined by Bei and Chiao [83], represents a customer’s assessment of the price they should pay for the product or service they receive. It includes how customers evaluate and make purchasing decisions based on price considerations [84]. Regarding sustainable clothing, price is often a barrier to purchase and consumption as these items often have higher price points compared to conventional clothing products [85]. However, Awuni et al. [86] argue that consumers are not necessarily deterred by the higher prices of sustainable products. Instead, they are willing to pay a premium for these items due to their positive environmental impact. This willingness reflects a broader trend where consumers are increasingly recognizing the value of sustainable products and are prepared to accept higher prices for their benefits.

Research suggests that perceived price positively influences environmental attitudes, as consumers understand the role of sustainable products in environmental protection and waste reduction [87]. Additionally, price has a significant impact on consumer attitudes and purchase intentions. Studies show that price affects how consumers perceive product quality and value [88]. For instance, research by Kopplin and Rösch [89] indicates that consumers are more inclined to purchase sustainable clothing when they perceive that its value and quality are commensurate with the price paid. This perception leads to greater satisfaction with the purchase and reinforces the consumer’s intention to buy green products. Based on these findings, the following hypotheses are proposed:

**H5:** Perceived price significantly affects environmental attitude.
**H6:** Perceived price significantly affects product attitude.

#### 2.2.4. Social Influence (SI) and consumer attitude

Social influence (SI) refers to the process by which individuals adjust their attitudes and behaviors to align with the expectations and behaviors of others, including individuals, groups, mass media, and commercial advertisements [90]. It encompasses the ways in which external factors, such as the attitudes and behaviors of peers, family, and social networks, shape an individual’s opinions, beliefs, and ultimately, their consumption behaviors [91]. Palomo-Domínguez et al. [92] note that Generation Z consumers exhibit great concerns regarding climate change and environmental degradation. This concern drives their interest in sustainable products that minimize environmental harm. Additionally, consumer choices are frequently shaped by the attitudes and endorsements of their social circle. Individuals are more likely to adopt a favorable attitude towards a product if it is endorsed by friends and acquaintances [93]. This phenomenon extends to sustainable clothing products, as evidenced by research indicating that consumers are more inclined to adopt sustainable clothing if their family or friends also use such products, leading to a positive attitude and intention to consume them [94].

Further reinforcing this idea, research by Maziriri et al. [95] underscores the significant role of social influence in shaping environmental attitudes. Their study reveals that when individuals are influenced by the environmental attitudes and behaviors of those around them, such as family and colleagues, they develop more favorable perceptions of environmental issues. This highlights the critical role social influence plays in promoting sustainable behaviors. Similarly, Purboyo et al. [28] found that both social influence and environmental perceptions significantly influence attitudes toward purchasing green products. These findings suggest that encouragement from family, friends, and the community can promote positive attitudes toward environmentally friendly products. Furthermore, an awareness of environmental issues and the benefits of green products contributes to consumers’ willingness to invest in these items. Therefore, social influence plays a crucial role in shaping consumer attitudes and behaviors towards sustainable consumption. Based on these findings, the following hypotheses are proposed:

**H7:** Social influence significantly affects environmental attitude.
**H8:** Social influence significantly affects product attitude.

#### 2.2.5. Product Design (PD) and consumer attitude

Product design (PD) involves the meticulous creation of comprehensive product descriptions tailored to meet customer requirements [96]. In the context of sustainable clothing, the challenge lies in balancing aesthetic appeal, functionality, and longevity. High-street clothes often suffer from issues such as poor quality and short lifespan despite their trendy designs [97]. To address this, a key approach is to visually communicate sustainability through product design by focusing on extending the lifespan of products [98].

Sustainable product design encompasses several critical elements, including aesthetic appeal, functionality, innovation, and adaptability to changing market trends. These designs often feature multifunctionality, user co-creation, and a commitment to longevity [99]. Research by Haase et al. [100] identifies three main factors that significantly influence product attitudes: aesthetics, function, and symbolism. Their study suggests that integrating these elements effectively, while appealing to the five human senses, enhances the overall product experience and fosters a positive evaluation.

Furthermore, designing products with positive environmental impacts—such as through eco-friendly characteristics, performance, pricing, eco-labels, and health attributes—also plays a crucial role in shaping environmental attitudes [101]. A well-designed product that balances aesthetic appeal and functionality while reducing environmental harm can significantly influence consumer attitudes toward sustainable clothing. Studies by van der Merwe [29] support this notion, demonstrating that consumers are inclined to favor products with sustainable designs, not only because they fulfill aesthetic desires but also because they represent a commitment to environmental protection. Therefore, product design plays a significant role in forming positive consumer attitudes towards sustainable clothing. Based on these findings, the following hypotheses are proposed:

**H9:** Product design significantly affects environmental attitude.
**H10:** Product design significantly affects product attitude.

#### 2.2.6. Environmental Concern (EC) and consumer attitude

Environmental concern (EC), often referred to as ecological effect, involves an individual’s emotional commitment to addressing environmental problems and threats [102]. This concept reflects the public’s awareness, capability, and engagement in tackling environmental challenges [103]. The rising interest in sustainable clothing production and consumption highlights the critical role of environmental concern, particularly in countries like China, where awareness is rapidly growing [104, 105].

Research consistently shows a positive correlation between high levels of environmental concern and a stronger inclination toward environmentally friendly consumption behaviors, including the purchase of sustainable products [106]. For instance, Leclercq-Machado et al. [49] found that Peruvian consumers who are highly concerned about environmental issues prefer sustainable clothing. Their attitudes, shaped by environmental concern, lead to a higher intention to purchase such products in an effort to reduce environmental impact. Moreover, Dhir et al. [107] observed that increased environmental concern positively influences environmental attitudes, which in turn affects product attitudes [108]. This relationship suggests that individuals who are deeply concerned about environmental issues are more inclined to cultivate favorable attitudes toward products that are environmentally friendly and possess a lower ecological footprint. This is supported by Whitmarsh and O’Neill [109], who found that individuals with strong environmental concern are more likely to engage in pro-environmental behaviors and express positive attitudes toward products that align with their ecological values. Additionally, Hartmann and Apaolaza [110] reinforce this perspective, demonstrating that consumers with high levels of environmental concern are more inclined to support and purchase products characterized by minimal ecological impact. Their findings emphasize that environmental concern is a significant driver in shaping both attitudes toward environmental issues and specific products. Based on these findings, it is clear that environmental concern plays a crucial role in influencing consumer attitudes towards sustainable products. Building upon these insights, the following hypotheses are proposed:

**H11:** Environmental concern significantly affects environmental attitude.
**H12:** Environmental concern significantly affects product attitude.

#### 2.2.7. Environmental Knowledge (EK) and consumer attitude

Environmental knowledge, as defined by Sun et al. [111], encompasses an understanding of issues and concepts related to the environment and ecosystem. Simanjuntak et al. [112] further elaborated on this notion by asserting that environmental knowledge also reflects individuals’ awareness of their impact on the surrounding environment. With the increasing awareness of environmental pollution caused by the textile industry, consumers are becoming more conscious of how sustainable clothing can help alleviate these adverse effects [113–115]. In recent years, the importance of environmental knowledge has increased among consumers of textile products [106, 116], particularly because the textile industry is recognized as the second most polluting sector after oil in many developing countries [7]. Therefore, enhancing consumers’ environmental knowledge regarding sustainable clothing is essential for fostering positive attitudes towards these products [117].

Research by Leclercq-Machado et al. [49] highlights that increasing consumers’ understanding of the environmental impact of human behavior leads to more favorable attitudes and a greater willingness to purchase sustainable products, including clothing. Environmental knowledge enables consumers to distinguish the characteristics and environmental impact of sustainable products from conventional products, thereby improving their attitudes towards sustainable products [118, 119]. Studies by Kumar [120], Malik and Singhal [121], and Leclercq-Machado et al. [49] demonstrate that environmental knowledge influences product attitude and significantly impacts environmental attitude. These findings further reinforce that consumers with a higher level of environmental knowledge are more likely to develop positive attitudes toward green products. Their enhanced awareness of environmental issues and the causes of pollution leads to greater overall appreciation and inclination towards sustainable consumption. In summary, environmental knowledge plays a significant role in shaping both environmental and product attitudes. Based on these insights, the following hypotheses are proposed:

**H13:** Environmental knowledge significantly affects environmental attitude.
**H14:** Environmental knowledge significantly affects product attitude.

#### 2.2.8. Environmental Attitude (EAT) and consumer attitude

Environmental attitude (EAT) encompasses an individual’s beliefs, feelings, and behavioral intentions regarding environmental issues [122]. As defined by Chen et al. [27], environmental attitudes represent “consumers’ evaluations of environmental protection efforts”. In essence, they reflect consumers’ positive reactions toward products that contribute to environmental sustainability. Environmental attitude plays a significant role in shaping consumer behavior, as individuals with stronger environmental concerns are more likely to engage in eco-friendly practices, including the purchase of sustainable clothing [52]. Research by Razzaq et al. [123] further supports this by demonstrating that consumers with a favorable environmental attitude tend to favor sustainable clothing. Individuals who are concerned about environmental protection prioritize consuming sustainably produced clothing to minimize their ecological footprint. The positive relationship between environmental attitude and purchase intention is well-documented. Consumers who hold strong environmental values are more inclined to purchase green products, reflecting their commitment to reducing environmental harm [27]. Furthermore, consumers who engage in ecologically friendly behaviors are more likely to engage in sustainable clothing consumption, as they perceive the ability to use sustainable clothing to help them reduce their negative environmental impact [124]. Thus, environmental attitudes not only influence specific behaviors, such as purchasing sustainable clothing, but also broadly affect intentions to buy environmentally friendly products. Therefore, the following hypothesis is proposed:

**H15:** Environmental attitude significantly affects purchase intention.

#### 2.2.9. Product Attitude (PAT) and consumer attitude

Product attitude (PAT), as defined by Luo and Zhong [125], is the cumulative result of various evaluations of brand attributes. It reflects consumers’ overall assessment of a product and plays a critical role in shaping their purchasing decisions. Previous research has consistently shown that product attitude significantly influences consumer behavior, particularly in relation to the intention to purchase green products [126, 127]. Given the rising awareness of environmental issues, particularly in light of the fashion industry’s substantial contribution to pollution, consumers increasingly prioritize eco-friendly options [2]. A recent study by Ahmed et al. [45] indicates that individuals who care about the environment and exhibit positive attitudes toward it are more likely to favor environmentally protective products. Notably, Generation Z has shown a pronounced interest in green products, valuing their environmentally friendly attributes [19]. In addition, research by Chen et al. [27] provides evidence that product attitude directly impacts purchase intention. They argue that when consumers perceive a product as environmentally beneficial, their positive attitudes towards it are likely to translate into higher purchase intentions. This underscores the importance of aligning product attributes with environmental benefits to enhance consumer willingness to buy green products. However, there exists a notable “attitude-behavior gap” wherein consumers may express favorable attitudes towards sustainable products without necessarily translating these attitudes into actual purchase behavior [48]. This gap often arises due to factors such as price, convenience, and perceptions of product quality. Understanding these factors is essential for manufacturers and retailers to develop strategies that effectively bridge this gap and encourage the purchase of sustainable products [49]. Based on these insights, the following hypothesis is proposed:

**H16:** Product attitude significantly affects purchase intention.

### 2.3. Research framework

**Fig 1:**
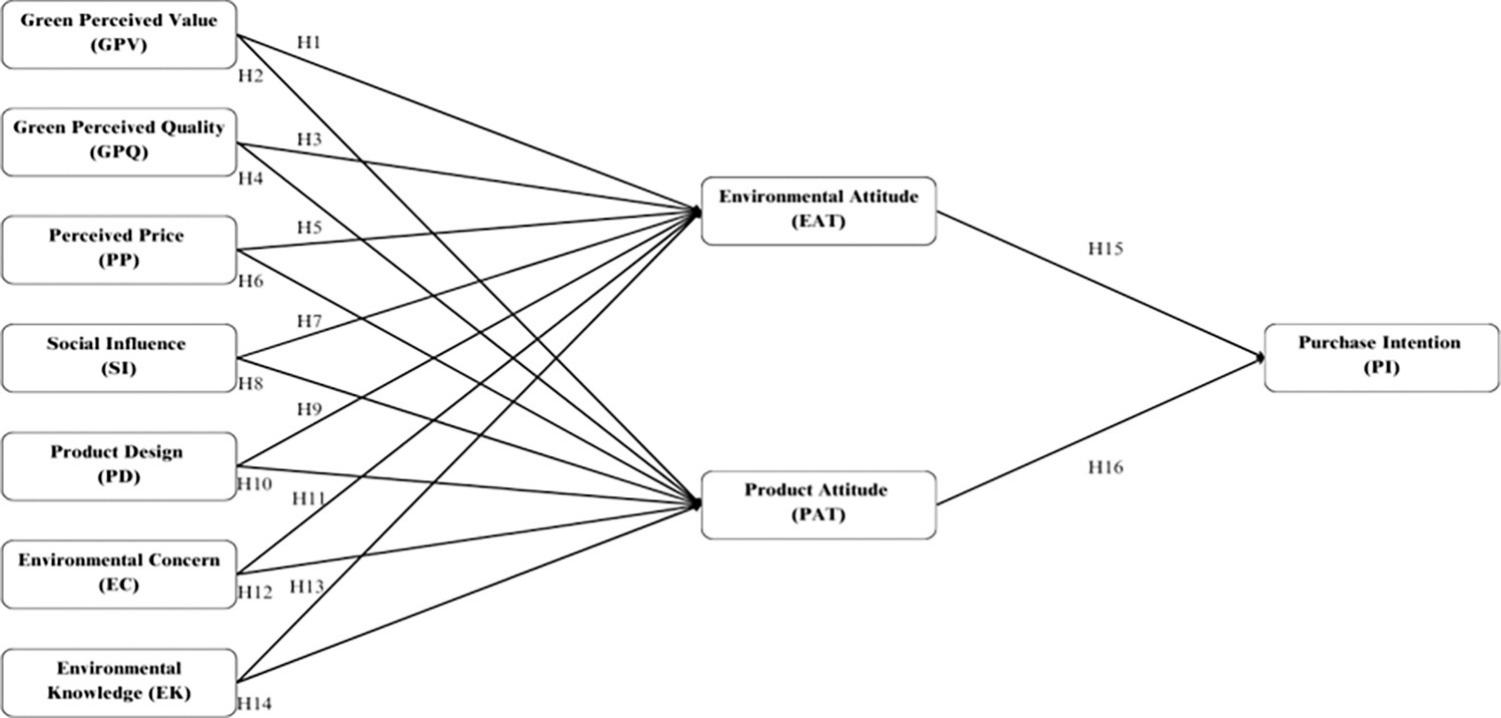
The proposed research framework.

The research framework has been constructed by integrating relevant literature and formulating hypotheses, as demonstrated in Fig 1, it investigates the impacts of Green Perceived Value (GPV), Green Perceived Quality (GPQ), Perceived Price (PP), Social Influence (SI), Product Design (PD), Environmental Concern (EC) and Environmental Knowledge (EK) as independent variables on the dependent variable Purchase Intention (PI). The mediating roles of Environmental Attitude (EAT), and Product Attitude (PAT) are also explored in these relationships.

## 3. Methodology

### 3.1. Research design

This study adopted a quantitative approach, comprising two main phases: a pilot study and an empirical study, aimed at ensuring the accuracy and reliability of the findings.

In the pilot study, a preliminary investigation was conducted to refine survey tools and confirm factors influencing Generation Z sustainable clothing purchasing behavior. Pilot studies are essential for ensuring the feasibility of the main [128]. Thirty participants were recruited for this phase, and their responses were used to assess the reliability of the questionnaire [129]. The Cronbach’s alpha value was set at a threshold of 0.7 to ensure questionnaire reliability [130]. Items with values below 0.7 were excluded from the finalized questionnaire to enhance its validity and effectiveness.

Following the pilot study, the empirical study involved a more extensive investigation using quantitative methods, facilitated through an online survey platform. The finalized questionnaire, refined based on insights gained from the pilot study, was employed for data collection. This phase aimed to test the research model and hypotheses proposed in the study. The research model underwent rigorous evaluation, and the hypotheses were systematically assessed using statistical analyses.

### 3.2. Instrument

The questionnaire was employed as the primary tool for quantitative data collection and was structured into three main sections. The first section outlined the study’s purpose, provided privacy assurances, and obtained participants’ consent. It also included essential definitions and explanations of sustainable clothing to ensure all respondents had a clear understanding of the concept. To assess familiarity, a screening question was included: “Have you ever purchased sustainable clothing products?”. This aimed to gauge prior exposure, but to further manage potential biases, an additional question was added to assess respondents’ knowledge of sustainable clothing “Can you identify specific sustainable clothing brands?”. This multi-step approach helped ensure that respondents not only had experience with sustainable clothing but also had adequate knowledge to participate meaningfully, enhancing the accuracy and reliability of the data.

The second section of the questionnaire focused on gathering demographic data such as gender, age, income, and assessing respondent’s sustainable clothing purchasing behavior. This demographic information facilitated audience classification, while detailed inquiries into shopping habits provide a comprehensive understanding of the respondent’s intentions to purchase sustainable clothing and the factors influencing these decisions.

The third section comprised 40 items meticulously designed to measure factors influencing the intention to purchase sustainable clothing. These items have been carefully adapted from previous studies, ensuring a reliable and robust foundation for the current investigation. This section is instrumental in understanding the various factors that influence the intention to purchase sustainable clothing, including GPV, GPQ, PP, SI, PD, EC, EK, EAT, PAT, PI. Drawing upon the research works of Albayrak et al. [131], Ansu-Mensah [132], Chen et al. [133], Chen et al. [27], Doszhanov and Ahmad [134], Dunlap et al. [135], Karell and Niinimäki [136], Kumar et al. [137], Lee [138], Qomariah and Prabawani [139], Surova [140], these factors were meticulously curated to ensure their relevance and applicability to the present study. Respondents rated these items on a five-point Likert scale, ranging from “Strongly Disagree” (1) to “Strongly Agree” (5). This comprehensive approach provided valuable insights into the decision-making process when purchasing sustainable clothing. This design ensured robust data collection, controlling for knowledge biases and providing meaningful insights into the variables influencing purchase intentions. The constructs and their corresponding items are presented in Table 2.

### 3.3. Participants

The study targeted Generation Z individuals in Vietnam with prior experience in purchasing and using sustainable clothing, encompassing those born between 1997 and 2012 [58]. Notably, participants aged 18 and above were included, ensuring the acquisition of relevant, reliable, and legal information. The survey included both male and female respondents from the Generation Z cohort. Out of 641 participants, 223 were male (34.8%) and 418 were female (65.2%). In terms of income distribution, a substantial proportion fell within the 10–15 million VND range (22.8%). Concerning purchasing frequency over three months, 499 respondents reported purchasing sustainable clothing less than five times (77.8%). Table 1 presents an overview of the demographic characteristics of the survey participants for this study.

**Table 1:** Respondents’ profile.

Respondents’ characteristics (N = 641)		Frequency	Percentage
**Gender**	Male	223	34.8
	Female	418	65.2
**Income**	Under 5 million VND	129	20.1
	From 5–10 million VND	134	20.9
	From 10–15 million VND	146	22.8
	From 15–20 million VND	114	17.8
	Over 20 million VND	118	18.4	
**Purchase frequency over 3 months**	Less than 3 times	247	38.5	
	From 3–5 times	252	39.3	
	More than 5 times	142	22.2	

### 3.4. Data collection

The research utilized a non-probability sampling technique, specifically convenience sampling, to collect data. This approach was chosen due to its practicality, efficiency, and cost-effectiveness [141], aligning well with the study’s scope and resource constraints. Convenience sampling facilitated rapid data collection and addressed logistical challenges encountered in the research environment [142].

Data collection was conducted through both online and offline channels to ensure a diverse and representative sample. Participants were recruited via email invitations and through in-person visits to educational institutions and offices. All individuals provided informed consent before participating in the study. To protect their privacy, the questionnaires were anonymized, and respondents were informed that they could withdraw from the study at any time if they felt uncomfortable. The study received ethical approval from FPT Can Tho University, Vietnam (Approval No. 20240402.02). The primary tool for data collection was a Google Form questionnaire, which was distributed directly in-person or through email and popular social media platforms, including Facebook, Zalo, and Instagram. The survey was conducted from April 5 to April 18, 2024, resulting in 641 valid responses. This sample size is considered suitable for statistical analysis, adhering to the rule of thumb suggested by Hair et al. [143], which recommends a minimum of ten times the number of scaling items.

### 3.5. Data analysis

Upon data collection, rigorous analysis was conducted using Statistical Package for the Social Sciences (SPSS) and Analysis of Moment Structures (AMOS) software [144]. Cronbach’s Alpha was initially employed to assess the reliability and consistency of the survey items. Subsequently, Exploratory Factor Analysis (EFA) was performed to validate the theoretical framework’s structure, ensuring internal reliability and appropriateness of the hypothesized factors and relationships. Confirmatory Factor Analysis (CFA) further confirmed the constructs included in the framework. In the main phase of statistical analysis, Structural Equation Modeling (SEM) was employed to examine the interrelationships among the factors proposed by the hypotheses. This technique integrates factor analysis with multiple regression analysis, providing a comprehensive understanding of the underlying relationships. To streamline the analysis, items were coded using the initials of the factors and their numerical order in the questionnaire (Table 2). After removing unsuitable variables, items were renamed accordingly to enhance clarity and interpretability.

**Table 2:** Reliability and EFA.

Factors/variables	Reliability	EFA		Convergence validity	
	Cronbach’s alpha	Eigen values	Factor loadings	CR	AVE
**Green Perceived Value (GPV)** [134]	0.807	1.677		0.873	0.633
GPV1: “I purchase sustainable clothing because it is environmentally friendly.”			0.787		
GPV2: “I purchase sustainable clothing because it has more environmental benefits than other products.”			0.746		
GPV3: “Purchasing sustainable clothing would make a good impression on others.”			0.748		
GPV4: “When I buy sustainable clothing products, I get value for its money.”			0.761		
**Green Perceived Quality (GPQ)** [133]	0.868	9.284		0.904	0.654
GPQ1: “The quality of sustainable clothing is regarded as the best benchmark with respect to environmental concern.”			0.760		
GPQ2: “The quality of sustainable clothing is reliable with respect to environmental consideration.”			0.767		
GPQ3: “The quality of sustainable clothing is durable with respect to environmental performance.”			0.758		
GPQ4: “The quality of sustainable clothing is excellent with respect to environmental image.”			0.787		
GPQ5: “The quality of sustainable clothing is professional with respect to environmental reputation.”			0.762		
**Perceived Price (PP)** [132, 139]	0.796	1.464		0.858	0.669
PP1: “Price is a major concern for me to go for sustainable clothing.”			0.793		
PP2: “The price of sustainable clothing is acceptable.”					
PP3: “Sustainable clothing has a fairly affordable price.”			0.780		
PP4: “I think the price of sustainable clothing is in line with the value of the product.”			0.766		
**Social Influence (SI)** [27]	0.825	2.116		0.884	0.656
SI1: “I learn from my friends, family and classmates about sustainable clothing products.”			0.753		
SI2: “If my friends purchase sustainable clothing products, I will buy them.”			0.772		
SI3: “I will share information on how to buy sustainable clothing products with my friends.”			0.794		
SI4: “Most of my friends and family buy sustainable clothing products.”			0.756		
**Product Design (PD)** [136, 140]	0.758	1.535		0.861	0.674
PD1: “The clothing has a classic design, aesthetically sustainable over time.”			0.778		
PD2: “The design of the clothing is optimal and can be recycled into other products.”			0.779		
PD3: “The design of sustainable clothing is simple but beautiful.”			0.776		
**Environment Concern (EC)** [131, 135, 138]	0.808	1.829		0.874	0.634
EC1: “Environmental protection will help people have a better quality of life.”			0.750		
EC2: “I am concerned about environmental development.”			0.780		
EC3: “I often think about the potential negative development of the environmental situation.”			0.751		
EC4: “I am concerned that humanity will cause lasting damage towards the environment.”			0.773		
**Environment Knowledge (EK)** [27]	0.778	1.594		0.871	0.692
EK1: “I know what sustainable clothing is.”			0.775		
EK2: “I know what an environmental label is.”			0.817		
EK3: “I know that using sustainable clothing reduces the damage to the environment.”			0.785		
**Environmental Attitude (EAT)** [27]	0.866	2.411		0.903	0.651
EAT1: “Advocating an environmentally friendly lifestyle is necessary.”			0.742		
EAT2: “I think the government needs to focus more on environmental protection.”			0.748		
EAT3: “It is very important to promote consumers’ attention to environmental issues.”			0.781		
EAT4: “I think it’s important to control environmental pollution.”			0.762		
EAT5: “I think the earth’s resources are limited, so environmental protection is important.”			0.745		
**Product Attitude (PAT)** [27]	0.828	1.954		0.886	0.659
PAT1: “I prefer using sustainable clothing products over other general clothing products.”			0.771		
PAT2: “I think purchasing sustainable clothing products is good for me.”			0.728		
PAT3: “I think sustainable clothing products that can reduce environmental damage are important.”			0.752		
PAT4: “I am willing to purchase sustainable clothing products that are good for the environment.”			0.760		
**Purchase Intention (PI)** [137, 139]	0.824	2.000		0.883	0.654
PI1: “I consider purchasing sustainable clothing.”			0.751		
PI2: “I intend to buy sustainable clothing instead of conventional clothing in the future.”			0.757		
PI3: “I might possibly buy sustainable clothing in the future.”			0.776		
PI4: “I would consider buying sustainable clothing if I happen to see them in a (n) (online) store.”			0.771		

Additionally, SmartPLS software was utilized to further assess discriminant validity, and to calculate R-squared (R^2^), Q-squared (Q^2^), and f-squared (f^2^), providing insights into the predictive accuracy, effect sizes, and overall model fit. This combination of tools ensured robust statistical analysis and reliable measurement of the proposed constructs.

## 4. Results

### 4.1. Reliability test and Exploratory Factor Analysis (EFA)

To assess the reliability of the scale in the research model and the correlation between observed variables and total variables [130], Cronbach’s alpha analysis was used. The factors were evaluated based on the criterion that Cronbach’s alpha coefficient should be greater than or equal to 0.7 [140]. Table 2 displays the results of the Cronbach’s alpha test for each factor in the model: GPV, GPQ, PP, SI, PD, EC, EK, EAT, PAT, and PI, revealing that all Cronbach’s alpha indices for the factors range from 0.758 to 0.868. These results indicate that the reliability of the questionnaire used in the study is acceptable.

Subsequently to determine the basic structure of a set of observed variables in quantitative research, the Exploratory Factor Analysis (EFA) method was employed [145]. During the initial analysis, item PP2 was excluded from consideration. Upon reanalysis, the data presented in Table 2 revealed 10 distinct factors with no overlap between them, indicating that the questions were constructed into a well-defined set of scales. Additionally, 10 factors were extracted with the Eigenvalue of 1.464 exceeding the threshold of 1, indicating high factor loading coefficients exceeding the threshold of 0.5 [146].

To assess the appropriateness of the EFA analysis, the standards outlined by Hair et al. were applied. The Kaiser-Meyer-Olkin (KMO) measure, which indicates sampling adequacy, was required to exceed 0.5, while the Bartlett test needed to show significance at the 0.05 level, corresponding to 95% confidence. The results of EFA revealed a KMO index of 0.906, exceeding the threshold of 0.5. Additionally, the Bartlett test produced a significance level lower than 0.05, confirming the statistical significance of the analysis. Furthermore, the Total Variance Explained (TVE) was calculated to be 66.321%, which exceeds the 60% benchmark recommended by Gerbing and Anderson [147], indicating that the extracted factors account for 66.3% of the total variance. This robust factor structure supports the reliability and validity of the measurement model used in this study.

### 4.2. Confirmatory factor analysis (CFA)

Confirmatory Factor Analysis (CFA) was conducted to assess the fit between the research data and the theoretical model, following the guidelines suggested by Kline [148]. Besides, CFA also helps provide convergent and discriminant validity values within the theoretical framework.

For the CFA model to be considered appropriate, it must satisfy specific criteria. These include a Chi-square/df ratio of less than 5, Tucker-Lewis Index (TLI) and Comparative Fit Index (CFI) values exceeding 0.9, Goodness of Fit Index (GFI) surpassing 0.8, Root Mean Square Error of Approximation (RMSEA) below 0.08, and Probability of Close Fit (PCLOSE) greater than 0.05, as proposed by Hu and Bentler [149]. Examination of the results presented in Table 3 reveals that the CFA model in the study meets these stipulated conditions, with Chi-square/df = 0.983, CFI = 1.000, TLI = 1.001, GFI = 0.951, RMSEA = 0.000, and PCLOSE = 1.000.

**Table 3:** Measurement model CFA.

Goodness-of-fit indices	Value Obtained	Recommend Value
Chi-square/df	0.983	< 5
GFI	0.951	> 0.8
TLI	1.001	> 0.9
CFI	1.000	> 0.9
RMSEA	0.000	< 0.08
PCLOSE	1.000	> 0.05

Moreover, the regression weight index of all variables in the model is greater than 0 and has a *p*-value < 0.001. Additionally, the standard regression weights of all variables in the model are also greater than 0.5, as recommended by Gerbing and Anderson [147].

To evaluate discriminant validity, two criteria were employed: Fornell and Larcker and Heterotrait Monotrait Ratio of Correlations (HTMT). According to Fornell and Larcker [150], the square root of the AVE for each construct must be greater than the correlation coefficients between constructs. The results in Table 4 confirm this criterion, as all diagonal elements (representing the square root of the AVE) are larger than the off-diagonal correlation coefficients, indicating that the model satisfies the Fornell and Larcker criterion for discriminant validity. Furthermore, discriminant validity was assessed using the HTMT criterion, as suggested by Henseler et al. [151]. HTMT values below 0.9 indicate satisfactory discriminant validity. Table 5 shows that all HTMT indicators are below the 0.9 threshold, further confirming that the model meets the discriminant validity requirements.

**Table 4:** Discriminant validity (Fornell and Lacker).

_EAT	EC	EK	GPQ	GPV	PAT	PD	PI	PP	SI
**EAT**	**0.807**									
**EC**	0.295	**0.797**								
**EK**	0.330	0.169	**0.832**							
**GPQ**	0.339	0.340	0.248	**0.809**						
**GPV**	0.320	0.203	0.224	0.310	**0.795**					
**PAT**	0.380	0.320	0.277	0.326	0.326	**0.812**				
**PD**	0.331	0.225	0.220	0.235	0.237	0.313	**0.821**			
**PI**	0.316	0.268	0.240	0.344	0.223	0.338	0.291	**0.809**		
**PP**	0.298	0.195	0.255	0.260	0.270	0.279	0.203	0.272	**0.818**	
**SI**	0.334	0.247	0.279	0.269	0.249	0.347	0.280	0.298	0.236	**0.810**

*Note*: GPV = Green Perceived Value; GPQ = Green Perceived Quality; PP = Perceived Price; SI = Social Influence; PD = Product Design; EC = Environmental Concern; EK = Environmental Knowledge; EAT = Environmental Attitude; PAT = Product Attitude; PI = Purchase Intention.

**Table 5:** Discriminant validity (HTMT).

_EAT	EC	EK	GPQ	GPV	PAT	PD	PI	PP	SI
**EAT**									
**EC**	0.350								
**EK**	0.397	0.208							
**GPQ**	0.387	0.403	0.303						
**GPV**	0.374	0.249	0.283	0.369					
**PAT**	0.447	0.389	0.341	0.379	0.398				
**PD**	0.405	0.288	0.285	0.291	0.299	0.395			
**PI**	0.373	0.327	0.304	0.406	0.266	0.405	0.368		
**PP**	0.366	0.249	0.334	0.322	0.344	0.352	0.267	0.346	
**SI**	0.393	0.300	0.346	0.317	0.303	0.419	0.351	0.362	0.298

*Note*: GPV = Green Perceived Value; GPQ = Green Perceived Quality; PP = Perceived Price; SI = Social Influence; PD = Product Design; EC = Environmental Concern; EK = Environmental Knowledge; EAT = Environmental Attitude; PAT = Product Attitude; PI = Purchase Intention.

### 4.3. Collinearity statistics (VIF)

This study used a self-response survey methodology that allows respondents to complete the survey independently. Although this approach offers convenience and efficiency, it has the potential to cause common method bias (CMB), which can occur when responses to different questions are overly similar, leading to highly correlated data and potential multicollinearity. To address this issue, the variance inflation factor (VIF) is used to measure the degree of multicollinearity between latent variables [152]. According to Hair et al. [153], a VIF value greater than 5 indicates problematic multicollinearity, while a more conservative threshold of VIF < 2 is often recommended in quantitative analysis. Some studies, such as Johnston et al. [154], suggest that VIF values exceeding 2.5 may indicate multicollinearity issues.

In this study, as shown in Table 7, the VIF values for the independent variables ranged between 1.167 and 1.296, well below the critical thresholds. These results confirm that multicollinearity is not a concern in this model, ensuring the validity and reliability of the analysis. The absence of multicollinearity also supports the discriminant validity of the constructs in the model.

### 4.4. Structural Equation Modeling (SEM) analysis

**Fig 2:**
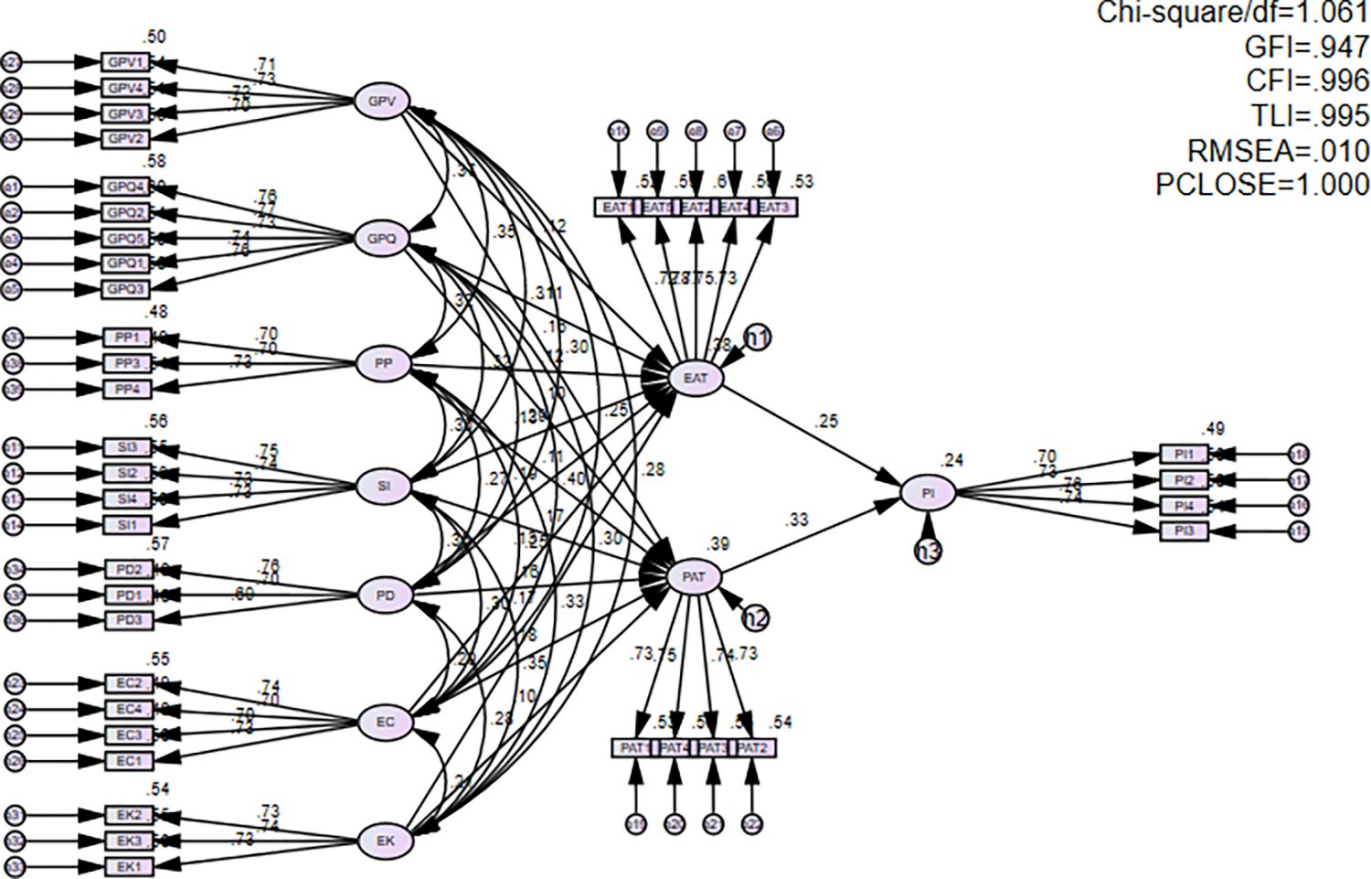
SEM model.

Structural Equation Modeling (SEM) is used to evaluate the correlation between components in the research model. The results, presented in Fig 2 and Table 6, indicate favorable fit indices, including Chi-square/df = 1.061, CFI = 0.996, TLI = 0.995, GFI = 0.947, RMSEA = 0.010, and PCLOSE = 1.000. These findings suggest that the model accurately describes the data, and the set of scales utilized in this study is suitable, as proposed by Byrne [155].

**Table 6:** Measurement model SEM.

Goodness-of-fit indices	Value Obtained	Recommend Value
Chi-square/df	1.061	< 5
GFI	0.947	> 0.8
TLI	0.995	> 0.9
CFI	0.996	> 0.9
RMSEA	0.010	< 0.08
PCLOSE	1.000	> 0.05

The results of the path coefficient analysis, as presented in Table 7, validate the acceptance of all proposed hypotheses. Each hypothesis demonstrates statistical significance with a sig value (*p*-value) of less than 0.05, aligning with the 95% confidence level. Specifically, the path from GPV to EAT exhibits a *p*-value of 0.009 and a path coefficient of 0.124, confirming the influence of GPV on EAT and thereby supporting H1. This finding suggests that heightened consumer recognition of the environmental benefits associated with sustainable clothing positively impacts their attitudes toward environmental conservation. Besides, the path from GPV to PAT shows a *p*-value of 0.001 and path coefficient of 0.157, confirming the influence of GPV on PAT and thereby supporting H2. This finding shows that consumers perceive greater value from using sustainable clothing products, leading to more positive attitudes towards them.

**Table 7:** Hypotheses testing results (direct relationships).

Hypotheses	Relationships	Path coefficients	*p*-values	Results	VIF
H1	EAT <—GPV	0.124	0.009	Accepted	1.206
H2	PAT <—GPV	0.157	0.001	Accepted	1.206
H3	EAT <—GPQ	0.114	0.017	Accepted	1.296
H4	PAT <—GPQ	0.101	0.040	Accepted	1.296
H5	EAT <—PP	0.118	0.016	Accepted	1.211
H6	PAT <—PP	0.108	0.032	Accepted	1.211
H7	EAT <—SI	0.130	0.006	Accepted	1.230
H8	PAT <—SI	0.174	***	Accepted	1.230
H9	EAT <—PD	0.187	***	Accepted	1.176
H10	PAT <—PD	0.162	0.001	Accepted	1.176
H11	EAT <—EC	0.125	0.007	Accepted	1.194
H12	PAT <—EC	0.175	***	Accepted	1.194
H13	EAT <—EK	0.174	***	Accepted	1.183
H14	PAT <—EK	0.100	0.041	Accepted	1.183
H15	PI <—EAT	0.249	***	Accepted	1.167
H16	PI <—PAT	0.335	***	Accepted	1.167

Note

*** *p* < 0.001

GPV = Green Perceived Value; GPQ = Green Perceived Quality; PP = Perceived Price; SI = Social Influence; PD = Product Design; EC = Environmental Concern; EK = Environmental Knowledge; EAT = Environmental Attitude; PAT = Product Attitude; PI = Purchase Intention.

Moreover, the path from GPQ to EAT exhibits a *p*-value of 0.017 and the path coefficient of 0.114, confirming the influence of GPQ on EAT and thereby supporting H3. This suggests that users tend to evaluate a product’s environmental performance, equating it with the quality of sustainable clothing products, thus contributing to their positive environmental attitudes and reputation for environmental responsibility. Additionally, the path from GPQ to PAT represents the *p*-value of 0.04 and the path coefficient of 0.101, confirming the influence of GPQ on PAT and thereby supporting H4. This finding indicates that consumers have a good attitude towards sustainable products when these products possess high quality and are associated with an environmentally friendly image.

Furthermore, the path from PP to EAT exhibits a *p*-value of 0.016, and the path coefficient of 0.118, confirming the influence of PP on EAT and thereby supporting H5. This finding shows that consumers perceive the price of sustainable clothing as commensurate with the value the product brings and they express a positive attitude towards the product. Similarly, the path PP to PAT exhibits the *p*-value of 0.032 and the path coefficient of 0.108, confirming the influence of PP on PAT and thereby supporting H6. This finding shows that consumers tend to perceive the prices of sustainable clothing products as reasonable when they align with the value these products offer.

Moreover, the pathways from SI to EAT and PAT indicate *p*-values of 0.006 and 0.000, respectively, with corresponding path coefficients of 0.13 and 0.174, confirming the influence of SI on both EAT and PAT and thereby supporting H7 and H8 respectively. This observation suggests that consumers exhibit a preference for sustainable clothing products when they observe similar choices within their social networks.

The path from PD to EAT displays a *p*-value of 0.000 and a path coefficient of 0.187, confirming the influence of PD on EAT and thereby supporting H9. This finding underscores that consumers prioritize environmental considerations and favor products designed with sustainability in mind. Similarly, the path from PD to PAT showcases a *p*-value of 0.001 and a path coefficient of 0.162, substantiating the influence of PD on PAT and thus supporting H10. This finding shows that consumers tend to choose and have a positive view of sustainable clothing products that are simple, have beautiful designs and bring value to them.

The path from EC to EAT has a *p*-value of 0.007 and a correlation of 0.125, which supports the effect of EC on EAT and thus supports H11. This review confirms that environmentally conscious consumers are more likely to engage in environmental practices such as choosing sustainable products such as clothing. Furthermore, the path from EC to PAT has a *p*-value of 0.000 and a path coefficient of 0.175, which supports the effect of EC on PAT, so supports H12. This review confirms that consumers are choosing sustainable clothing products because of their commitment to environmental protection.

The path from EK to EAT manifests a *p*-value of 0.000 and a path coefficient of 0.174, affirming the influence of EK on EAT and thereby supporting H13. This finding indicates that individuals possessing comprehensive environmental knowledge tend to hold more favorable attitudes toward green products and the environment. This heightened awareness empowers consumers to make informed decisions, opting for sustainable clothing products that align with their environmental values. Besides, the pathway from EK to PAT represents a *p*-value of 0.041 and a path coefficient of 0.1, confirming the impact of EK on PAT and thereby supporting H14. This finding suggests that consumers are capable of developing a favorable attitude towards environmentally friendly products, such as sustainable clothing.

On the other hand, the path from EAT to PI exhibits a *p*-value of 0.000. and the path coefficient is 0.249, confirming the influence of EAT on PI and thereby supporting H15. This observation indicates that consumers exhibit a heightened intention to purchase sustainable clothing products when they recognize the importance of environmental protection and harbor a stronger inclination to make purchases. Additionally, the pathway from PAT to PI represents a *p*-value of 0.000 and a path coefficient of 0.335, confirming the impact of PAT on PI and thereby supporting H16. This finding underscores that consumers are inclined to elevate their intention to purchase sustainable clothing products when they perceive personal benefits and environmental harm mitigation associated with the product.

In summary, the research findings confirm that GPV, GPQ, PP, SI, PD, EC, EK, significantly influence EAT and PAT, which in turn influence purchase intention (PI) among Generation Z consumers in Vietnam. Both EAT and PAT serve as important mediators between these factors and PI. However, while GPQ positively influences both EAT and PAT, the mediation of PAT in the relationship between GPQ and PI is weaker, with a lower path coefficient (0.101) and a p-value of 0.040. This indicates that while product quality impacts attitudes, its effect on purchase intention through PAT is not as strong as other factors. In contrast, PAT plays a significant role in mediating the effects of other factors like GPV, SI, and EC on PI.

### 4.5. Evaluation of explanation and prediction power of research model

To evaluate the explanatory and predictive power of the research model, R-squared and Q-squared indices are used. The Squared Multiple Correlations coefficient (R-squared) explains the impact of independent variables on the dependent variable and measures the overall power of the model [156]. Additionally, the f-squared index measures the influence of independent variables on the dependent variable [152]. In models with multiple variables, each variable may have its own R-squared index, reflecting its role as either an intermediate or dependent variable. This study focuses on three variables that act as dependent variables: EAT, PAT, and PI.

The results indicate that the PAT variable has the highest R-squared value of 0.39 (39%). This suggests that approximately 39% of the variation in PAT is explained by the variation among the independent variables GPQ, GPV, PP, SI, PD, EC, and EK. The R-squared index for EAT is 0.383, indicating that 38.3% of the variation in EAT is explained by the same set of independent variables. Finally, the R-squared index for PI is 0.238, showing that 23.8% of the change in PI is due to changes in the correlation among the independent variables.

Regarding the f-squared index, the results show that the f-squared values for the independent factors influencing EAT range from 0.014 to 0.028. Specifically, the f-squared values are as follows: GPV is at 0.018, GPQ at 0.017, PP at 0.014, SI at 0.019, PD at 0.028, EC at 0.015, and EK at 0.028. Notably, both EK and PD exhibit f-squared indices greater than 0.02, suggesting that these factors exert a more significant influence on EAT compared to the other independent variables. For PAT, the f-squared values for the independent variables range from 0.01 to 0.029, with GPV at 0.024, GPQ at 0.012, PP at 0.010, SI at 0.029, PD at 0.022, EC at 0.026, and EK at 0.010. These findings indicate that GPQ, PP, and EK have a relatively minimal impact on PAT when compared to the other factors. Furthermore, the f-squared indices for EAT and PAT affecting PI are 0.049 and 0.066, respectively, indicating that both EAT and PAT contribute significantly to explaining PI.

Regarding the out-of-sample predictive power (Q-squared), this index measures the model’s predictive ability. According to Shmueli et al. [156], Q-squared values greater than 0 demonstrate that the values of the variables built in the model are consistent with reality, thus confirming the model’s predictive ability. The results of this study show that all Q-squared indexes are greater than 0, specifically EAT (0.184), PAT (0.179), and PI (0.098). These values indicate the model’s effectiveness in prediction and show a close connection between model predictions and actual observations.

In summary, the model demonstrates strong explanatory and predictive power, with significant portions of the variance in EAT, PAT, and PI being explained by the independent variables. This underscores the robustness and reliability of the model in understanding the factors influencing these variables.

## 5. Discussion

This study investigates the key determinants influencing purchase intention among Vietnamese Generation Z consumers regarding sustainable clothing. The research findings validate the influence of various independent factors, namely Green Perceived Value (GPV), Green Perceived Quality (GPQ), Perceived Price (PP), Social Influence (SI), Product Design (PD), Environmental Concern (EC), and Environment Knowledge (EK) on the Purchase Intention (PI) of Generation Z regarding sustainable clothing in the Vietnamese context, with Environmental Attitude (EAT) and Product Attitude (PAT) serving as mediating factors.

The study identifies consumer perception-related factors, including GPV, GPQ, and PP, as significant drivers of both EAT and PAT. The findings confirm that GPV positively impacts EAT (hypothesis H1), consistent with findings from studies by Chen and Chang [73] and Sheth et al. [74]. This relationship suggests that consumers’ recognition of the environmental benefits associated with sustainable clothing enhances their sense of environmental responsibility and encourages positive attitudes toward environmental protection. Sangroya and Nayak [157] further emphasize this functional value by highlighting the economic and practical benefits that green products provide, reinforcing positive attitudes. In today’s context, modern consumers are increasingly motivated to seek sustainable solutions, not only to safeguard the planet but also to enhance their personal quality of life [158]. Thus, consumers are driven to choose products that minimize ecological impacts while offering practical benefits, such as health and convenience. This shift reflects a broader societal trend where choosing sustainable products is intertwined with the aspiration to foster a better future amidst pressing environmental challenges. Additionally, the result showed a significant positive impact of GPV on PAT (hypothesis H2), indicating that heightened awareness of the environmental benefits of sustainable clothing positively influences consumer attitudes toward these products. This aligns with findings from Liao et al. [30] and Ahn and Kwon [159], who argue that GPV fosters both positive attitudes and stronger intentions to purchase sustainable fashion. The implication is that GPV not only informs consumers about the eco-friendly aspects of sustainable clothing but also contributes to a positive self-image, reflecting a lifestyle dedicated to environmental stewardship. Moreover, the research findings indicate a significant positive effect of GPQ on EAT (hypothesis H3), consistent with Ahmad et al. [160] findings. Consumers tend to evaluate a product’s environmental performance, equating it with the quality of sustainable clothing products, which reinforces their positive environmental attitudes and reputation for environmental responsibility. In the wake of the fashion industry’s substantial environmental footprint, there is a growing expectation for consumers, particularly among Generation Z, to be more responsible in their consumption choices [2, 18, 19]. Sustainable clothing, produced through methods that minimize environmental harm and utilize organic materials, resonates with these consumers’ values. By recognizing the GPQ factor, consumers can actively demonstrate their commitment to environmental and social responsibility through their purchasing choices. Additionally, GPQ significantly influences PAT (hypothesis H4), in line with findings from Chen et al. [82]. When consumers perceive a product’s quality as sustainable, they tend to develop favorable attitudes toward it. The perception of quality is critical, particularly as many sustainable products leverage advanced technologies and high-quality materials, which enhances perceived value and justifies a higher price point. This tendency indicates that consumers are often willing to pay a premium price for products that they believe provide both environmental and personal benefits. Interestingly, the study found that PP significantly impacts EAT (hypothesis H5), which diverges from findings by Chekima et al. [161]. This suggests that, contrary to some perspectives, higher prices do not necessarily deter Generation Z’s environmental attitudes within the Vietnamese context. This divergence may be attributed to the socioeconomic profiles of the samples; our research targets a relatively affluent Generation Z, while Chekima et al. [161] included a broader range of income levels in Malaysia. This highlights the necessity of understanding the contextual nuances that shape consumer behavior regarding sustainability. Furthermore, this study findings indicate that PP also significantly influences PAT (hypothesis H6), supported by Rodrigues et al. [88]. This suggests that consumers tend to perceive the prices of sustainable clothing products as reasonable when they align with the value these products offer. This suggests that price is not evaluated in isolation; rather, consumers consider long-term benefits such as quality, durability, and the positive societal impacts of their purchases. In a climate where environmental pollution is a critical concern, sustainable clothing is increasingly recognized as a viable solution to mitigate environmental impacts. Although these products may carry a higher price tag, the intangible values—such as ethical production and environmental preservation—render them a worthy investment for conscious consumers.

The results underscore the significant influence of SI on both EAT and PAT (hypotheses H7 and H8), consistent with findings from Chen et al. [90] and Maziriri et al. [95]. Consumers’ attitudes and behaviors are heavily shaped by their social circles, as individuals often adopt the values and practices of those around them. When consumers are surrounded by peers who prioritize sustainability, they are more likely to cultivate an environmentally conscious mindset [162]. This phenomenon, known as normative social influence, reveals how social contexts can create pressure to conform to sustainable behaviors, encouraging individuals to favor sustainable clothing when they see friends and family making similar choices [91]. However, while social influence plays a critical role, it also raises questions about the depth of these attitudes. Are individuals genuinely committed to sustainability, or are they merely conforming to perceived social norms? The superficial adoption of sustainable practices can lead to what some researchers call “performative sustainability”, where individuals engage in eco-friendly behaviors primarily for social approval rather than a genuine commitment to environmental values. This highlights the need for marketers to not only leverage social influence but also to foster deeper environmental awareness and commitment among consumers.

Additionally, the study reveals that PD significantly impacts both EAT and PAT (hypotheses H9 and H10). This finding is consistent with the research conducted by Marcon et al. [101] and Surova [140], which emphasizes the importance of sustainable product design in shaping consumer attitudes. Consumers increasingly favor products that prioritize environmental considerations, demonstrating a preference for sustainable clothing characterized by versatility, recyclability, and minimal adverse environmental impacts. This aligns with a growing consumer trend towards sustainable aesthetics, where clothing designs that are trendy, minimalist, and visually appealing resonate more with eco-conscious shoppers. However, the emphasis on aesthetics raises critical questions about consumer motivation. Are consumers choosing sustainable clothing merely for its appearance, or do they understand and appreciate its environmental benefits? The allure of well-designed, sustainable products may enhance consumer interest, but it is essential for brands to communicate the underlying environmental values effectively. This approach will not only reinforce positive attitudes but also deepen consumer engagement with sustainability. By integrating sustainability into product design, businesses can effectively improve consumer attitudes and foster a greater willingness to engage in sustainable consumption practices. The findings underscore the importance of emphasizing sustainable design attributes in marketing strategies. Companies that prioritize eco-friendly designs can differentiate themselves in a competitive marketplace, appealing to environmentally conscious consumers while promoting positive attitudes toward sustainable products. However, brands must remain vigilant against the risk of greenwashing—where companies exaggerate or misrepresent their sustainability efforts—as this could erode consumer trust and commitment over time.

Furthermore, EC and EK emerge as critical determinants of both EAT and PAT. The study finds that EC significantly influences EAT (hypothesis H11), fostering positive consumer attitudes towards environmental protection. This aligns with the findings of Dhir et al. [107] and is further supported by Klerk et al. [106], which demonstrate that individuals with heightened EC are more likely to engage in environmentally friendly behaviors, such as choosing sustainable products. As the world confronts severe environmental challenges, including climate change, there is a growing imperative for consumers to care about environmental issues [1]. Increasing awareness has led more individuals to support environmental protection efforts, particularly in the context of the fashion industry, which is recognized as a major contributor to ecological harm [2, 18, 19]. In this light, sustainable clothing plays a crucial role in mitigating environmental damage, especially for consumers with a strong sense of environmental responsibility who are inclined to adopt sustainable consumption practices. Similarly, EC significantly influences PAT (hypothesis H12), consistent with the research of Eid and El-Gohary [163]. This suggests that consumers prefer sustainable clothing products because they contribute positively to environmental protection [108]. This positive perception is attributed to the durable and recyclable attributes of sustainable clothing, which not only serve environmental goals but also provide tangible benefits to consumers. On the other hand, EK significantly impacts EAT (hypothesis H13), as supported by Dhir et al. [107], Goh and Balaji. [119], and Pinto et al. [102]. While challenging to quantify, environmental knowledge is essential for fostering responsible consumption behaviors [164]. This underscores the pivotal role of environmental knowledge in driving responsible consumption behaviors. A well-informed consumer is more likely to recognize the importance of environmental protection and subsequently develop favorable attitudes towards sustainable products. By enhancing their understanding of the environmental consequences of their consumption choices, individuals become more inclined to support environmentally friendly options, including sustainable clothing. Moreover, EK also exerts a significant influence on PAT (hypothesis H14), as evidenced by Kumar [120] and Malik and Singhal [121]. These findings underscore that individuals with a comprehensive understanding of environmental issues tend to hold more positive attitudes toward green products. This awareness empowers consumers to make informed decisions, ultimately leading to the adoption of sustainable clothing products that resonate with their environmental values.

The study findings reveal that both EAT and PAT act as crucial mediators in influencing the intention to purchase (PI) sustainable clothing. This underscores how consumer concern for sustainability affects perceptions of environmental impact and shapes preferences for sustainable clothing products, ultimately guiding purchase intentions. Firstly, the significant impact of EAT on PI (hypothesis H15) is consistent with the research conducted by Chen et al. [27]. This is further supported by Stern et al. [165], who examined the psychological responses of consumers to environmental concerns and their direct influence on green purchasing intentions. These studies collectively suggest that consumers are more likely to intend to purchase sustainable clothing when they recognize the importance of environmental protection. This indicates that cultivating positive environmental attitudes can be a powerful driver of green consumption behavior. Secondly, PAT also significantly influences PI (hypothesis H16), as indicated by Chen et al. [27]. This suggests that consumers are more inclined to increase their intention for sustainable clothing when they perceive personal benefits and environmental harm mitigation associated with these products. Factors such as superior quality, durability, and appealing design not only reinforce consumers’ understanding that sustainable clothing protects the environment but also promise long-term savings, thereby intensifying the motivation to purchase. Additionally, the awareness that sustainable clothing is produced through environmentally friendly practices fosters a sense of contribution to a positive movement, further enhancing consumers’ commitment to sustainable consumption.

## 6. Implication

### 6.1. Theoretical implications

This study contributes to the existing literature on consumer purchase intention regarding sustainable clothing products by simultaneously examining the influence of multiple independent factors. Building upon the insights of Sun and Willson [166], which propose the formation of two attitudes preceding the intention to consume green products general attitude (environmental attitude) and specific attitude towards a particular product (product attitude) this study explores the concurrent impact of independent factors: green perceived value, green perceived quality, perceived price, social influence, product design, environmental concern, and environmental knowledge on both environmental attitude and product attitude. Furthermore, this study investigates the roles of environmental attitudes and product attitudes as mediators among independent variables and the dependent variable purchase intention. The integration of these variables within an analytical framework provides a profound understanding of their interrelationships, with implications for both academic research and practical applications. Notably, perceptions of green value and quality play pivotal roles in shaping both environmental attitude and product attitude, while perceived price influences consumer decision-making. Social influence also proves significant, as it impacts environmental and product attitudes through social networks, where sustainable consumption trends or environmental movements can effectively spread via online platforms, creating positive social pressure that encourages behavior change. Moreover, the study highlights the importance of product design, emphasizing its role not only aesthetically but also in fostering green lifestyles and environmental awareness. Environmental concerns and environmental knowledge significantly influence both environmental attitude and product attitude. The findings of this study underscore that a consumer’s specific attitude towards a product (product attitude) exerts a greater influence on consumption intention compared to the general attitude (environmental attitude). This suggests that when consumers have a clear positive perception of a product’s features, quality, and value, they are more likely to make a purchase decision. Overall, the insights derived from this study serve as a foundation for further research in this field, offering valuable implications for the academic sector.

### 6.2. Managerial implications

The apparel industry has made notable strides toward sustainability in recent years, but this research highlights an imbalance in the industry’s development and consumer perception. There remains a challenge in effectively communicating sustainability initiatives without risking consumer confusion, disinterest, or suspicion. Simultaneously, consumer interest in sustainable clothing has yet to reach a level where sustainability becomes a top priority or drives action to pressure the industry toward greater responsibility. Nevertheless, there’s been a notable uptick in interest among Generation Z in recent years, indicating that sustainability issues are becoming more salient to consumers. There is a growing trend where sustainable fashion is perceived as a status symbol in society [167], albeit concerns about environmental impacts during production also influence consumer behavior toward sustainability [168].

To address these challenges, several key managerial implications emerge: First, enhancing green perceived value is crucial. Marketers must emphasize the environmental benefits of sustainable clothing to raise consumer awareness and attitudes toward eco-friendly products. This can be achieved through targeted marketing campaigns, where the positive environmental impacts of each purchase are clearly highlighted, combined with emotionally engaging stories to create a deeper connection with consumers. Second, prioritizing green perceived quality is essential. Manufacturers should ensure that sustainable clothing products not only meet quality standards but also convey value to both the environment and consumers. This not only encourages consideration in purchasing decisions but also instills a sense of reassurance for consumers. Providing certifications, guarantees, or transparent information about the production process can enhance trust and affirm the quality of the products. In this way, consumers will feel more satisfied with their decisions, while also encouraging them to become advocates for the brand. Third, effective communication of perceived price and value perception is vital. Marketing strategies should focus on educating consumers about the true value of sustainable clothing, thereby encouraging them to develop positive attitudes toward the environment and be willing to accept higher price points. Marketing messages should emphasize the long-term benefits that sustainable products offer, such as durability and cost savings over time. This will help consumers recognize that the initial investment can be offset by the value of use and the positive environmental impact. Consequently, they will feel more confident in their purchasing decisions. Fourth, leveraging social influence is important. Understanding social dynamics and the role of influencers can help marketers grasp consumer perceptions and preferences, thereby developing targeted marketing campaigns that have a profound impact. Collaborating with influencers who advocate for sustainability not only enhances the ability to convey messages but also broadens the reach to a wider audience. Through these relationships, brands can build trust and authenticity, encouraging consumers to actively engage in the sustainable consumption movement. Fifth, emphasizing product design is crucial. Deep insights into product design preferences can guide marketers in creating appealing and trendy sustainable clothing options that resonate with consumers and drive adoption. Additionally, involving consumers in the design process through mechanisms such as feedback or co-creation initiatives can enhance engagement and connection. As a result, this approach not only creates products that align with consumer needs and desires but also builds a community around the brand, encouraging loyalty and long-term support. Sixth, harnessing environmental concerns is necessary. Communication strategies should highlight the environmental benefits of sustainable clothing, aligning with consumer awareness of environmental issues and motivating purchase intentions. Regularly updating consumers on efforts and progress in sustainability not only helps them feel more connected to the brand but also encourages responsible consumption. In this way, brands can create a positive consumer community where each individual feels they are contributing to a more sustainable future. Lastly, promoting environmental knowledge is key. By disseminating information about environmental sustainability, marketers can raise consumer awareness and understanding, fostering excitement and engagement with sustainable clothing products. Developing educational content, such as workshops, online courses, or visual resources, can empower consumers to make more informed choices. Through this approach, consumers not only become more knowledgeable but also actively contribute to promoting sustainability within their communities.

In summary, this study sheds light on essential factors influencing purchase intention for sustainable clothing among Generation Z consumers, offering valuable insights for marketers. By leveraging these insights, marketers can effectively connect with Generation Z, refine their marketing strategies, and achieve success in targeting this demographic segment.

## 7. Limitations and recommendation

Although this study contributes to the advancement of theoretical understanding and provides practical insights, it also faces certain limitations. First, the focus on Generation Z consumers in Vietnam limits the generalizability of the findings to other cultural contexts. Future research should broaden the scope by including participants from various regions and countries to gain a more comprehensive understanding of sustainable clothing consumption behaviors across diverse cultures. Second, this study did not clearly consider the differences between demographic groups such as gender, age, and income of consumers in the context of sustainable clothing consumption in Vietnam. Future studies should investigate the differences in sustainable clothing consumption intentions between men and women, and the differences between different age groups and different incomes to gain deeper insights into consumer behavior. This will help marketers to develop different marketing strategies that are suitable for each customer segment. Third, although this study examines factors influencing purchase intention, it does not address the gap between intention and actual behavior. Intentions may not always translate into real purchasing actions. Future research should explore actual purchasing behaviors, potentially incorporating moderators like availability, convenience, and social norms to better understand what drives sustainable clothing purchases. Additionally, with the rapid growth of Vietnam’s fashion industry and the rise of both domestic and international brands, building strong brand equity is essential for distinguishing brands in a competitive market. As AlSaleh [169] notes, establishing brand equity fosters consumer trust and satisfaction across various sectors. Future studies could explore factors influencing brand equity for sustainable apparel, such as brand image and brand awareness, as a means of helping sustainable brands stand out and succeed. Furthermore, this study solely focuses on sustainable clothing products, and thus, the conclusions drawn may not be universally applicable to other product categories. Future research should explore and test models for various specialized green products such as green restaurants, hotels, and energy-efficient products to gain a more nuanced understanding of consumer behavior in diverse contexts. Addressing these limitations in future research endeavors can enhance the credibility and applicability of the findings, foster theoretical development, and assist marketers in devising more targeted and effective strategies to engage consumers.

## 8. Conclusion

The objective of this study is to investigate the key factors influencing Generation Z’s intention to purchase sustainable clothing in Vietnam, focusing on the mediating roles of Environmental Attitude (EAT) and Product Attitude (PAT). This study employed analytical techniques including Cronbach’s alpha, Exploratory Factor Analysis (EFA), Confirmatory Factor Analysis (CFA), and Structural Equation Modeling (SEM) to assess the research model and its associated hypotheses, with a sample of 641 participants. The results revealed that all seven factors—Green Perceived Value (GPV), Green Perceived Quality (GPQ), Perceived Price (PP), Social Influence (SI), Product Design (PD), Environmental Concern (EC), and Environmental Knowledge (EK)—significantly impact purchase intentions through EAT and PAT.

The study found that among external factors, Product Design (PD) had the strongest influence on Environmental Attitude (EAT), highlighting the importance of attractive and high-quality sustainable products in shaping consumer attitudes. Additionally, Green Perceived Value (GPV) and Green Perceived Quality (GPQ) positively influenced purchase intentions, demonstrating that when consumers recognize the environmental benefits and superior quality of sustainable products, they are more likely to choose them. Perceived Price (PP) also played an important role, with consumers favoring products they perceive to offer value for money. Social Influence (SI) emerged as a key factor, with peer and family endorsements driving sustainable clothing purchases. Environmental Concern (EC) and Environmental Knowledge (EK) further reinforced purchase intentions, indicating that individuals with greater environmental awareness and knowledge are more inclined to adopt sustainable consumption behaviors. More importantly, the study highlights the crucial roles of both Environmental Attitude (EAT) and Product Attitude (PAT) in mediating the relationship between these factors and Purchase Intention (PI). A positive Environmental Attitude (EAT) enhanced consumer perceptions of the environmental value of sustainable products, which in turn strengthened Product Attitude (PAT). Consumers with favorable attitudes toward both the environment and sustainable products were more likely to develop strong purchase intentions. This underscores the importance of fostering positive attitudes as they directly influence consumers’ likelihood of engaging in sustainable consumption.

This research provides important insights for marketers looking to appeal to Generation Z, emphasizing the need to highlight the environmental benefits, quality, and design of sustainable clothing products. By addressing these factors and fostering greater environmental awareness, businesses can effectively drive the shift toward more sustainable consumption patterns.

## Supporting information

S1 File Survey questionnaire. (PDF)

S2 File Dataset used in analysis. (XLSX)
