# Supplementary material for: Factors influencing Generation Z’s intention to purchase sustainable clothing products in Vietnam
Source: PLoS One. 2024 Dec 11;19(12):e0315502. doi: 10.1371/journal.pone.0315502 (PMC11634014; doi:10.1371/journal.pone.0315502)
Supplement: S1 File — (PDF) [file pone.0315502.s001.pdf]

QUESTIONNAIRE

Factors influencing Generation Z's intention to purchase sustainable clothing products in Vietnam.

This survey explores what factors influence Generation Z's intention to purchase sustainable clothing. We are interested in understanding factors such as green perceived value, green perceived quality, perceived price, social influence, product design, environmental concern, environmental knowledge and environmental attitude, how product attitude affects the intention to buy sustainable clothing.

We greatly appreciate your assistance in completing this survey. Your participation in this survey is completely voluntary and anonymous. We assure you that all the information you provide will be kept confidential and used only for research purposes.

Do you agree to participate in this survey and give consent for using your information for research purposes?

- o Yes
- o No

Sustainable clothing: *has been defined as clothing that "incorporates one or more aspects of social and environmental sustainability, such as Fair-Trade manufacturing or fabric containing organically-grown raw material" (Goworek et al., 2012).*

Have you ever purchased sustainable clothing products?

- o Used to
- o Never

Can you identify specific sustainable clothing brands? ________

Part 1: Demographics

1.1 Gender:

- o Male
- o Female
- o Other

1.2 Year of Birth:

- o 1965-1980
- o 1980-1996

- 1997-2006

1.3 What is your education level?

- High school
- Intermediate level
- College
- University
- Post graduate

1.4 What is your job?

- Student
- Teacher
- Business
- Office staff
- State officials and employees
- Freelancer
- Other:_____

1. 5 How much is your monthly income?

- Under 5 million
- From 5 - 10 million
- From 10 - 15 million
- From 15 - 20 million
- Over 20 million

Part 2: Information about shopping for sustainable clothing products

2.1 How do you usually buy sustainable clothing products?

- Buy directly at stores
- Buy online on e-commerce platforms such as Shopee, Lazada, Tiki

2.2 How much have you spent on sustainable clothing products over the last 3 months?

- Under 1 million
- From 1-2 million
- Over 2-4 million
- Over 4-6 million
- Over 6 million

2.3 How often do you buy sustainable clothing products over the last 3 months?

- Less than 3 times
- From 3 to 5 times
- More than 5 times

Part 3: Constructs and Items

The following section measures factors that influence purchasing intentions for sustainable clothing products. Please use a five-point Likert scale (e.g., 1 = strongly disagree, 2 = disagree, 3 = neutral, 4 = agree, 5 = strongly agree) for each statement.

**Green perceived value** (Doszhanov & Ahmad, 2015)	1	2	3	4	5
1) I purchase sustainable clothing because it is environmentally friendly.					
2) I purchase sustainable clothing because it has more environmental benefits than other products.					
3) Purchasing sustainable clothing would make me a good impression on others.					
4) When I buy sustainable clothing products, I get value for its money.					
**Green perceived quality** (Y. S. Chen et al., 2014)					
1) The quality of sustainable clothing is regarded as the best benchmark with respect to environmental concern.					
2) The quality of sustainable clothing is reliable with respect to environmental consideration.					
3) The quality of sustainable clothing is durable with respect to environmental performance					
4) The quality of sustainable clothing is excellent with respect to environmental image.					
5) The quality of sustainable clothing is professional with respect to environmental reputation.					
**Perceived price** (Adapted from Ansu-Mensah, 2021; Qomariah & Prabawani, 2020)					
1) Price is a major concern for me to go for sustainable clothing.					
2) The price of sustainable clothing is acceptable.					
3) The sustainable clothing has a fairly affordable price.					

4) I think the price of sustainable clothing is in line with the value of the product.					
**Social influences** (Chen et al., 2018)					
1) I learn from my friends, family and classmates about sustainable clothing products					
2) If my friends purchase sustainble clothing products, I will buy them					
3) I will share information to buy sustainable clothing products with my friends.					
4) Most of my friends and family buy sustainable clothing products.					
**Product design** (Karell & Niinimä ki, 2020; Surova, 2021)					
1) The clothing has a classic design, aesthetically sustainable over time.					
2) The design of the clothing is optimal and can be recycled into other products.					
3) The design of sustainable clothing is simple but beautiful.					
**Environmental Concern** (Albayrak et al., 2013; Dunlap et al., 2000; Lee, 2008)					
1) Environmental protection will help people have a better quality of life					
2) I am concerned about environmental development.					
3) I often think about the potential negative development of the environmental situation.					
4) I am concerned that humanity will cause lasting damage towards the environment.					
**Environmental knowledge** (Chen et al., 2018)					
1) I know what sustainable clothing is.					

2) I know what an environmental label is.					
3) I know that using sustainable clothing reduces the damage to the environment.					
**Environmental Attitude** (Chen et al., 2018)					
1) Advocating an environmentally friendly lifestyle is necessary.					
2) I think the government needs to focus more on environmental protection.					
3) It is very important to promote consumers' attention to environmental issues.					
4) I think it's important to control environmental pollution.					
5) I think the earth's resources are limited, so environmental protection is important.					
**Product Attitude** (Chen et al., 2018)					
1) I prefer using sustainable clothing products over other general clothing products.					
2) I think purchasing sustainable clothing products is good for me.					
3) I think sustainable clothing products that can reduce environmental damage are important.					
4) I am willing to purchase sustainable clothing products that are good for the environment.					

**Purchase Intention** (Kumar et al., 2017; Park & Lin, 2020)					
1) I consider purchasing sustainable clothing.					
2) I intend to buy sustainable clothing instead of conventional clothing in the future.					
3) I might possibly buy sustainable clothing in the future.					
4) I would consider buying sustainable clothing if I happen to see them in a(n) (online) store.					

Thank you very much for your support!
